# Supplementary material for: Patterns of Intron Gain and Loss in Fungi
Source: PLoS Biol. 2004 Nov 30;2(12):e422. doi: 10.1371/journal.pbio.0020422 (PMC532390; doi:10.1371/journal.pbio.0020422)
Supplement: Table S1 — Also available at http://genes.mit.edu/NielsenEtAl/. (4.3 MB ZIP). [file pbio.0020422.st001.zip › NielsenEtAl/html/345.html]

AN0066.1.NCU07884.1.MG06405.1.FG05414.1


```
 CLUSTAL W (1.82) Multiple Sequence Alignments - Introns Inserted


Sequence 1: MG06405.1	307 aa
Sequence 2: FG05414.1	387 aa
Sequence 3: NCU07884.1	309 aa
Sequence 4: AN0066.1	299 aa
Alignment Length: 387 aa
Number Identitical Residues: 176 aa
Alignment Score (without introns) 8546


MG06405.1 	--------------~--------------~------------------------------
NCU07884.1	--------------~--------------~------------------------------
FG05414.1 	MHPESESVSYCLGE1NPYLFGTLASGTSF0FLTSVEWPIVEISSLARALRPFRDLIPSRL
AN0066.1  	--------------~--------------~------------------------------
          	                                                            

MG06405.1 	-------------------MSTTLG2AFIAGGIAACGAVTATHPFETVKIR2MQLQGELR
NCU07884.1	-------------------MSTTAG2AFIAGGIAACGAVTATHPFETVKIR~MQLQGELQ
FG05414.1 	YILLLATDSRLLVPGELGAMSTTVG2AFIAGGIAACGAVTATHPFETVKIR2MQLQGELQ
AN0066.1  	-------------------MPFTS-~----------ALVALVQILIAVAPS~LQLQGELQ
          	                   *. *             . *: .: : :*    :******:

MG06405.1 	DKGHQPHFYRGPFHGVSVIVRNEGLGGIYRGISCA~YVYQILLNGCRLGFYEPMRQTLTG
NCU07884.1	SSTSSPHFYRGPIHGVSVIVRNEGLGGIYRGIGCA~YIYQILLNGCRLGFYEPMRHALAT
FG05414.1 	NKGHQPHHYRGPIHGVSVIVRNEGVRKIYRGIGAA~YIYQILLNGCRLGFYDPMRNALAK
AN0066.1  	AKSEAVKKYKGVLHGVKVILQNEGPRGLFRGIGSA0YIYQVLLNGCRLGFYEPIRNGLTT
          	 .    : *:* :***.**::***   ::***..* *:**:**********:*:*: *: 

MG06405.1 	LFYNDKNQQNLGINIFCGASSGIIGAAAGSPFFLVKTRLQSYSPFLPVGTQHEYKNALDG
NCU07884.1	TLFNDAKTQNLGINMFAGAASGIMGAMAGSPFFLAKTRLQSYSPFLPVGTQHQYKNAWDG
FG05414.1 	FIFKDGNAQNLGINMFCGAASGVIGAAAGSPFFLVKTRLQSFSKFRPVGTQHHYTGAWHG
AN0066.1  	AVYNDSHVQSLGINVFAGASSGIIGAAAGSPFFLVKTRLQSFSPFLPVGTQHQYRNSFDG
          	 .::* : *.****:*.**:**::** *******.******:* * ******.* .: .*

MG06405.1 	MRQIYGAEGAKGMYRGVGAAMIRTGFGSSVQLPTYFFAKRRLVKHLGMEEGPALHLASST
NCU07884.1	LTKIYKGEGVKGLYRGVGAAMIRTGFGSSVQLPTYFFAKRRLVKHLGMEEGPALHLASST
FG05414.1 	FKSIYSTEGIGGLYRGVQAAMIRTAFGSSVQLPTYFFAKRRLVRHFGMEEGPGLHLASSA
AN0066.1  	LRKIFASEGVGGLYRGVGAAMVRTGFGSSVQLPTYFFAKRRLVKHLGMEDGPALHLASST
          	: .*:  **  *:**** ***:**.******************:*:***:**.******:

MG06405.1 	VSGFVVCCVMHPPD1TIMSRMYNQSGNLYSGVLDCFAKTVKTEGVFAIYKGFLPHLARIL
NCU07884.1	ASGFVVCVVMHPPD1TIMSRLYNQNGNLYKGVFDCLAKTIRAEGFFAIYKGVIPHLTRIL
FG05414.1 	ISGFVVCCVMHPPD1TVMSRMYNQNGNLYTSAADCFAKTIRSEGIFALYKGFFPHLARIL
AN0066.1  	ASGFVVCCVMHPPD1TIMSRMYNQTGNLYKGVFDCLAKTIRTEGLFAIYKGYFAHLARIL
          	 ****** ****** *:***:***.****... **:***:::**.**:*** :.**:***

MG06405.1 	PHT~ILTLSLAEQTNKLMRRLEDHILPTEAL---
NCU07884.1	PHT~ILTLSLAEQTTKLVKKLEDKILGPETRVL-
FG05414.1 	PHT~ILTLSLAEQTNKLVRKIEGRILPSRMDMAT
AN0066.1  	PHT0ILTLSLAEQTNKLMRRVEDRLLSDSLKSRL
          	*** **********.**::::*.::*
```
